# Mangiferin Ameliorates Hyperuricemic Nephropathy Which Is Associated With Downregulation of AQP2 and Increased Urinary Uric Acid Excretion

**DOI:** 10.3389/fphar.2020.00049

**Published:** 2020-02-07

**Authors:** Xuechen Li, Zhenxin Yan, Mattias Carlström, Jinying Tian, Xiaolin Zhang, Wenxuan Zhang, Song Wu, Fei Ye

**Affiliations:** ^1^Beijing Key Laboratory of New Drug Mechanisms and Pharmacological Evaluation Study, Institute of Materia Medica, Chinese Academy of Medical Science & Peking Union Medical College, Beijing, China; ^2^Department of Physiology and Pharmacology, Karolinska Institutet, Stockholm, Sweden

**Keywords:** mangiferin, hyperuricemia, nephropathy, AQP2, uric acid excretion

## Abstract

Hyperuricemia is characterized by abnormally high level of circulating uric acid in the blood and is associated with increased risk of kidney injury. The pathophysiological mechanisms leading to hyperuricemic nephropathy (HN) involve oxidative stress, endothelial dysfunction, inflammation, and fibrosis. Mangiferin is a bioactive C-glucoside xanthone, which has been exerting anti-inflammatory, anti-fibrotic, and antioxidative effects in many diseases. This study aimed to evaluate the effect of mangiferin treatment in HN. In a mouse model of HN, we observed lower circulating urate levels and ameliorated renal dysfunction with mangiferin treatment, which was associated with reduced renal inflammation and fibrosis. We next investigated the mechanism of urate lowering effect of mangiferin. Metabolic cage experiment showed that mangiferin-administrated mice excreted significantly more urinary uric acid due to elevated urine output, but no marked change in urine uric acid concentration. Expressions of water channels and urate transporters were further assessed by western blot. Renal AQP2 expression was decreased, yet urate transporters URAT1, GLUT9, and OAT1 expressions were not affected by mangiferin in HN mice. Moreover, mangiferin treatment also normalized xanthine oxidase and SOD activity in HN mice, which would decrease uric acid synthesis and improve oxidative stress, respectively. Therefore, our results reveal a novel mechanism whereby mangiferin can reduce serum uric acid levels by promoting AQP2-related urinary uric acid excretion. This study suggested that mangiferin could be a multi-target therapeutic candidate to prevent HN *via* mechanisms that involve increased excretion and decreased production of uric acid and modulation of inflammatory, fibrotic, and oxidative pathways.

## Introduction

Hyperuricemia is one of the major causes of gout, due to circulating urate accumulation and crystallization. Moreover, experimental and epidemiological studies suggest that hyperuricemia is also a risk factor or predictor in the progression of several diseases, including hypertension (Richette and Bardin, 2010), cardiovascular diseases (Feig et al., 2008), metabolic syndrome (Ciarla et al., 2014), and chronic kidney disease (CKD) (Jalal et al., 2013; Johnson et al., 2013), although this is not supported by the results of Mendelian randomization studies (Jordan et al., 2019). Experimental studies showed that high level of uric acid activated oxidative stress, inflammation, fibrosis pathway, and induced kidney dysfunction (Jalal et al., 2013).

Excessive production and/or less excretion of uric acid are two major causes leading to the development of hyperuricemia. As the key enzyme in uric acid formation, xanthine oxidase (XO) catalyzes the oxidation from hypoxanthine to xanthine, then from xanthine to uric acid. During these enzymatic reactions, reactive oxygen species (ROS) are generated as a byproduct, inducing oxidative stress when accumulated (Harrison, 2004). It has been reported that XO activity was increased under hyperuricemia condition, activating oxidative stress pathway and causing kidney injury (Boban et al., 2014). These studies demonstrated that XO played a vital role in HN by both catalyzing uric acid generation and inducing oxidative stress. The selective XO inhibitors febuxostat and allopurinol are currently used in clinic to treat hyperuricemia and associated complications.

Two thirds of circulating uric acid undergoes renal excretion while the remaining one third is excreted in feces (Ichida et al., 2012). In the kidney, urate is freely filtered in the glomerulus, and about 90% is reabsorbed into the systemic circulation through urate transporters, and the remaining part is excreted in the urine (de Oliveira and Burini, 2012). Among the multiple urate transporters participating in urate handling in kidney, URAT1, located in the apical membrane of the proximal tubules, is an essential protein that mainly recognizes urate as substrate (Hosoyamada et al., 2004). Located in the apical and basolateral membrane of the proximal tubules, GLUT9 is another important urate transporter that reabsorbs uric acid in the kidney. Although also considered as a fructose transporter, genetic polymorphism of GLUT9 accounts for about 5% of serum uric acid variance (Vitart et al., 2008). In addition, OAT1 as another transporter expressed in the proximal tubules facilitates urate efflux from epithelial cells to the interstitium (Nigam et al., 2015). Currently, clinically used uricosuric drugs include benzbromarone, probenecid, and lesinurad. Use of these medications down-regulates urate transporters URAT1 or GLUT9 expression, promoting uric acid excretion (Tan et al., 2016; Tan and Miner, 2017). Although still a good choice for gout patients, use of benzbromarone caused liver toxicity in several cases (Kaufmann et al., 2005). Graded dosage increase was suggested to lower the incidence (Lee et al., 2008). Short half-life of probenecid limited its clinical use (Melethil and Conway, 1976). Selective URAT1 inhibitor lesinurad is suggested to be administrated in combination with an XO inhibitor to achieve the best medical effectiveness (Pan and Kong, 2016). When treating hyperuricemic nephropathy (HN), it would be desirable to have a drug that reduces the synthesis and increases the renal excretion of circulating uric acid, and have direct therapeutic effect on kidney injury, such as inflammation, fibrosis, and oxidative stress.

There are already some compounds that have dual effect in the kidney. For instance, Arhalofenate, which exhibited both XO inhibitive effect and anti-inflammatory effect (Stamp et al., 2018). Mangiferin is a natural C-glucoside xanthone that commonly exists in young leaves and bark of mango trees (Telang et al., 2013). Mangiferin was reported to lower serum uric acid levels (Niu et al., 2012; Yang et al., 2015) and exerts anti-inflammatory, anti-fibrotic, and antioxidative effects in diseases including diabetic nephropathy, acute kidney injury, NAFLD (He et al., 2014; Pal et al., 2014; Wang et al., 2017). These findings brought us the interest to investigate the effect of mangiferin on hyperuricemic nephropathy (HN). Moreover, the principal metabolite of mangiferin in the intestinal tract exhibited strong inhibition of XO (Niu et al., 2016).

The aim of the current study was to evaluate whether mangiferin could be a novel multi-target candidate to treat HN and explore the possible mechanisms. Based on these previous findings, we hypothesized that mangiferin might protect against HN through multiple pathways.

## Materials and Methods

### Animals and Experimental Procedures

Male ICR mice (18–22 g) were purchased from the Animal Center of Institute of Laboratory Animal Sciences, Chinese Academy of Medical Science, and Peking Union Medical College (CAMS & PUMC), Beijing, China. The mice had free access to water and regular rodent chow were housed under controlled temperature (21–23 °C) and humidity (40–60%) in a constant 12 h light/dark cycle. All animal experiments were carried out in accordance with the guidelines established by the National Institutes of Health for the care and use of laboratory animals and were approved by the regional Animal Care Committee of CAMS & PUMC.

Acclimated mice were randomly divided into four groups, i.e. control group (Con), mangiferin-treated control group (Con+Mang), hyperuricemic nephropathy model group (HN), and mangiferin-treated hyperuricemic nephropathy group (HN+Mang). Mangiferin (50 mg/kg/day, 0.1 ml/10 g body weight) was administrated 7 days in advance to model induction and also the following 10 days of induction by gavage. HN mice were induced by 300 mg/kg hypoxanthine (0.1 ml/10 g body weight) by gavage and 300 mg/kg oteracil potassium (0.2 ml/10 g of body weight, suspended in 0.5% sodium carboxymethyl cellulose solution) by subcutaneous injection (Meng et al., 2017) for 10 constitutive days. Negative control mice received vehicle. Metabolic cage experiment was performed at the last day of the induction. Mice had free access to water and chow in metabolic cages. The total 24 h urine output volume (*V*) was determined and aliquots were frozen for later analysis of uric acid levels (*uUA*) and total 24 h urinary uric acid excretion (*uUAex*) calculated as *uUAex* = *uUA* × *V*. Orbital blood samples were collected and the mice were euthanized to collect tissues for later analyses.

### Chemicals and Regents

**Figure:**
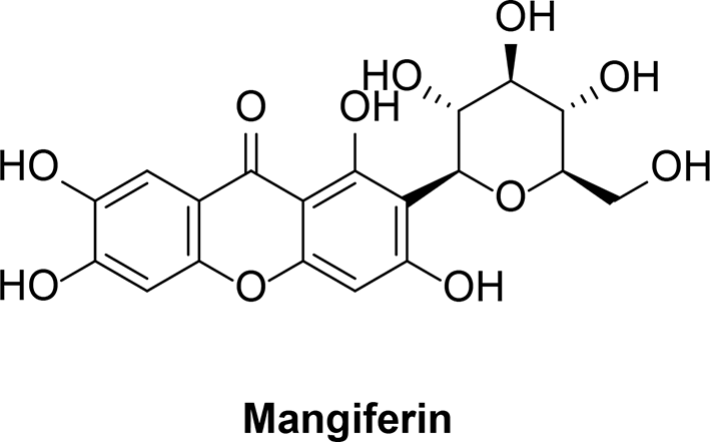


Mangiferin was purchased from the Innochem (Beijing, China). The purity was measured by HPLC-MS: 97.5%. The structure of mangiferin was determined by ^1^H NMR and MS spectrum. ^1^H NMR (400 MHz, DMSO-*d*_6_) δ 13.76 (s, 1H), 10.68 (s, 1H), 10.57 (s, 1H), 9.79 (s, 1H), 7.38 (s, 1H), 6.87 (s, 1H), 6.38 (s, 1H), 5.06–4.23 (m, 1H), 4.05 (t, *J* = 9.1 Hz, 1H), 3.75–3.65 (m, 1H), 3.41 (dd, *J* = 11.8, 5.8 Hz, 1H), 3.26–3.09 (m, 3H), ESI MS [M+Na]^+^ = 445.3.

AQP2, α-SMA, NLRP3, p-JNK, JNK, and OAT1 antibodies were purchased from Cell Signaling Technology (Danvers, MA, USA), URAT1, AQP1, p-PKCβI (Thr 641), and PKCβI antibodies were purchased from Santa Cruz Biotechnology (Dallas, TX, USA). GLUT9 antibody was purchased from Abcam (Cambridge, MA, USA). Commercial kits for detecting uric acid, creatinine, BUN, SOD, and XO were purchased from Nanjing Jiancheng Bioengineering Institute (Nanjing, China). RIPA buffer, ECL reactions were purchased from Applying Technologies Inc. (Beijing, China).

### Determination of Uric Acid, Creatinine, BUN, XO, and SOD Levels

Blood samples were centrifuged at 3000 × g for 4 min to obtain serum. Levels of serum, urine, kidney uric acid, creatinine, BUN, XO, and SOD were measured using commercial standard kits accordingly with the manufacturer’s protocol.

### Histopathology Staining

Mice kidneys were fixed with 4% paraformaldehyde for 24 hours, paraffin-embedded, and sectioned into 5μm-thick slices for further H&E, Masson’s trichrome and F4/80 staining and assessed by light microscopy. F4/80 positive brown staining was analyzed by Image-pro plus 6.0 (Media Cybernetics, Inc., Rockville, MD, USA) by an independent company Servicebio (Wuhan, China) in a blinded way (i.e. without any knowledge about the experimental groups).

### Western Blot Analysis

Whole kidney tissues were cut and homogenized in ice-cold RIPA buffer, followed by ultrasonication. The lysates were placed on ice for 30 min and centrifuged at 12,000 × g for 10 min. The supernatant was collected and protein levels were measured by BCA assay. Equal amount of protein was mixed with 5 × loading buffer. Samples were boiled at 100 °C for 5 min and cooled on ice for 5 min, then stored at −80 °C for further use.

Protein samples were separated by 10% SDS-PAGE and then transferred to PVDF membranes. The membrane was blocked by 2% BSA for 1 h at room temperature and then incubated with primary antibodies diluted at 1:1000 overnight in 4 °C. The secondary antibodies were incubated at room temperature for 1 h, at dilution of 1:5000. Then the membranes were applied electrogenerated chemiluminescence and analyzed by gel image analysis system (Flurochem 5500, Alpha Innotech, USA).

### Statistical Analysis

All the data was presented as means ± *SD and* analyzed by one-way or two-way ANOVA, followed by appropriate posthoc test, using Graphpad Prism6 software. *P* value less than 0.05 were considered statistically significant.

## Results

### Mangiferin Lowered Serum Uric Acid Level and Alleviated Renal Dysfunction in Hyperuricemic Mice

**Figure 1:**
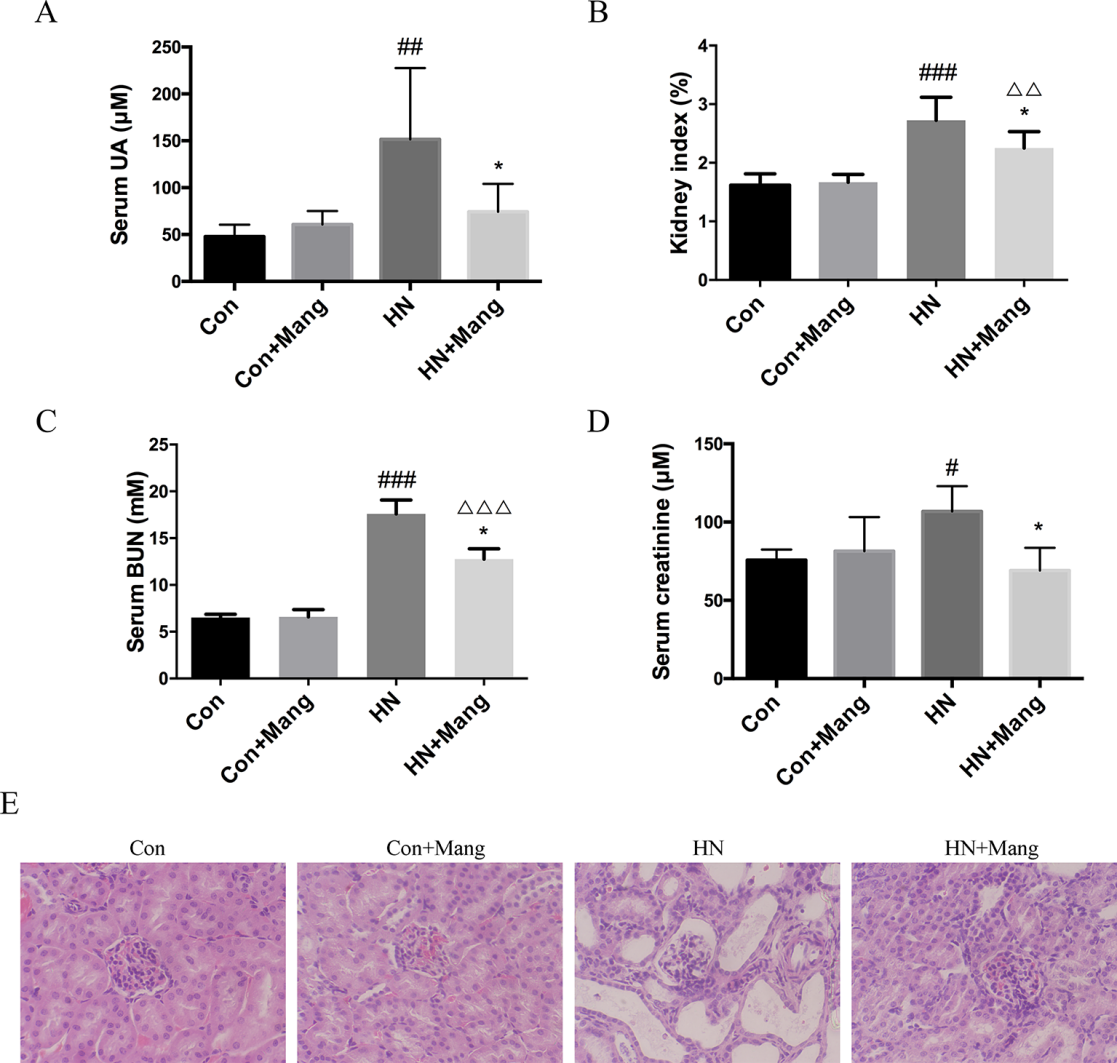
Effects of mangiferin on uric acid level and renal injury. Serum uric acid was measured **(A)**. Kidney injury was evaluated by calculated kidney index **(B)**, level of serum creatinine **(C)**, and BUN **(D)**. Kidney sections were applied to H&E staining (200×) **(E)**. n = 4-6. ^#^*P* < 0.05, ^##^*P* < 0.01, ^###^*P* < 0.001 vs. Con; *P < 0.01 vs. HN; ^△△^*P* < 0.01, ^△△△^*P* < 0.001 vs. Con+Mang.

HN mice were induced by oteracil potassium and hypoxanthine. Using this model, serum uric acid level was increased by more than 60% compared with that of control mice (**Figure 1A**). Markers of kidney dysfunction and injury, including kidney/body weight ratio (kidney index), serum BUN, and creatinine levels were all found to be elevated in mice treated with oteracil potassium and hypoxanthine (**Figures 1B–D**). Moreover, H&E staining of kidney sections showed massive glomerular hypertrophy and tubular dilation in hyperuricemic mice, indicating severe glomerular and tubular injury (**Figure 1E**).

In this HN model, simultaneous treatment with mangiferin prevented the increase in serum uric acid level, attenuated the kidney index, reduced serum BUN levels, and normalized serum creatinine levels (**Figures 1A–D**). Besides, mangiferin improved glomerular and tubular structures (**Figure 1E**). Meanwhile, mangiferin did not have any significant effects in normal control mice. Taken together, in an established and validated model of HN, mangiferin treatment was associated with several renal protective effects.

### Mangiferin Reduced Renal Inflammation in HN Mice

**Figure 2:**
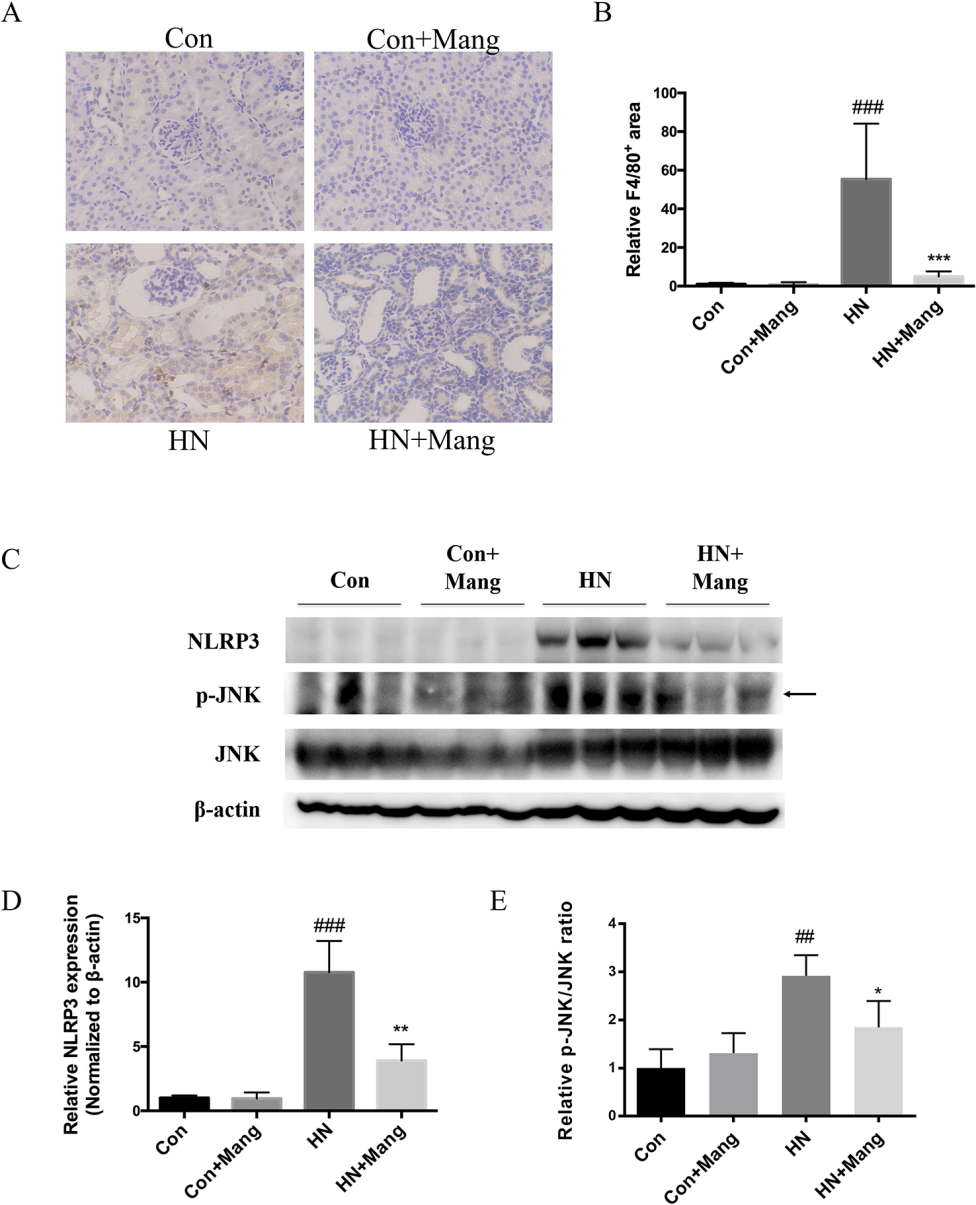
Protective effect of mangiferin on renal inflammation. F4/80 staining was applied to present macrophage infiltration in kidney (200×) **(A)**. The positive brown-stained area was calculated **(B)**. Expression of NLRP3, p-JNK and JNK was determined by western blot **(C)** and quantifications **(D)**. ^##^*P* < 0.01, ^###^*P* < 0.001 vs. Con; ^*^P < 0.05, **P < 0.01, ***P < 0.001 vs. HN.

Activation of the immune system and subsequent infiltration of inflammatory cells into the kidney is crucially involved in the pathology of kidney injury. We performed immunohistochemical staining of F4/80 to assess macrophage infiltration of the kidney. F4/80 positive staining area was significantly increased in HN mice compared with that in control mice (**Figures 2A, B**). These inflammatory changes were markedly reduced by mangiferin. There was no distinction at F4/80 staining in control mice with or without mangiferin administration.

We then measured inflammation-related protein expression and marker of apoptosis. NLRP3 expression was increased in HN mice and was reversed by mangiferin, as well as the ratio of p-JNK/JNK (**Figures 2C–E**). These results suggest anti-inflammatory and cell protective effects of mangiferin in the kidney of HN mice.

### Mangiferin Suppressed Renal Fibrosis in HN Mice

**Figure 3:**
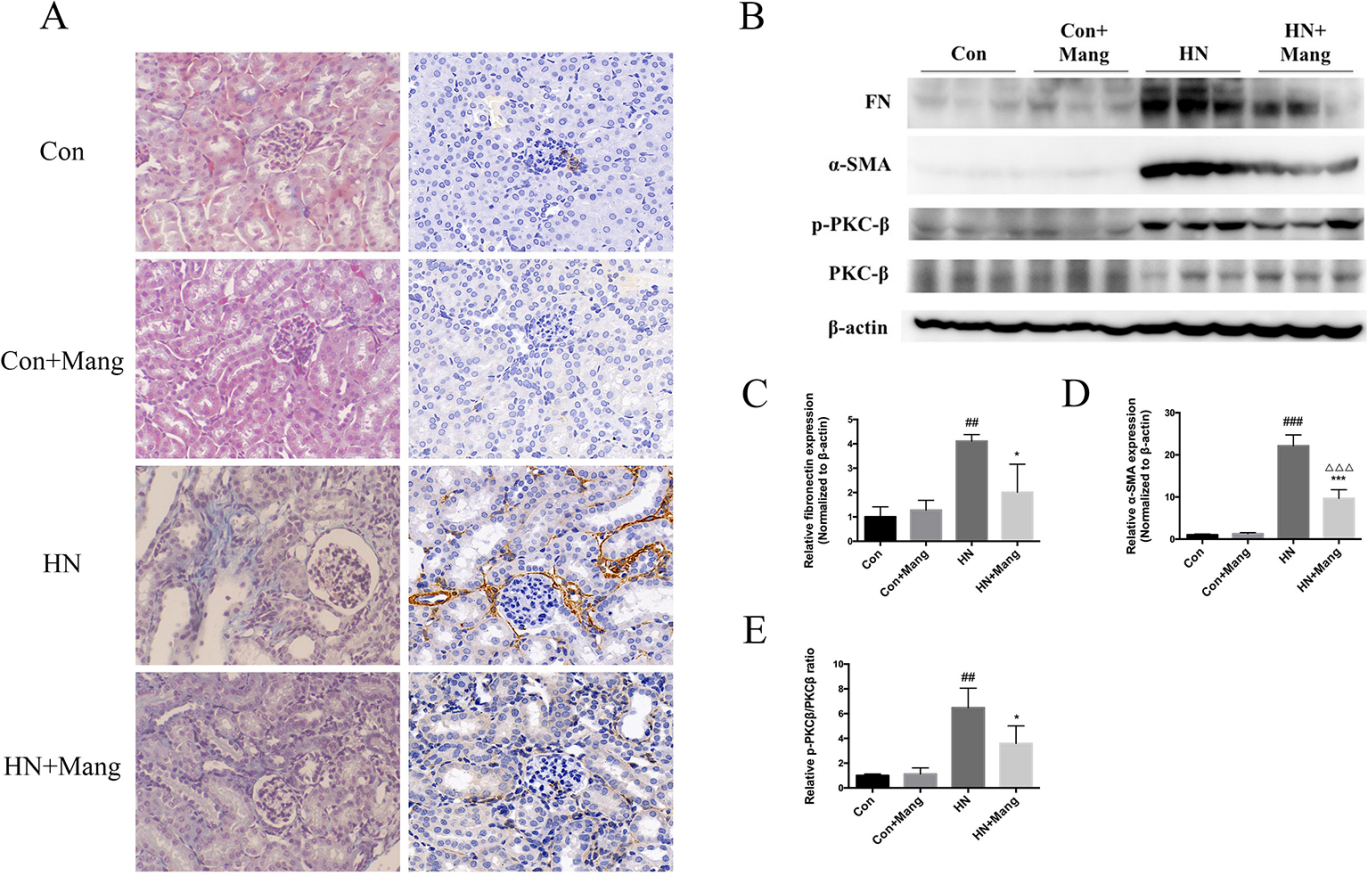
Protective effect of mangiferin on renal fibrosis. Kidney sections were applied to Masson’s trichrome stain (200×) (left) and α-SMA staining (200×) (right) **(A)**. Expression of fibronectin, α-SMA, p-PKCβ, and PKC-β was determined by western blot **(B)** and quantifications **(C–E)**. ^##^*P* < 0.01, ^###^*P* < 0.001 vs. Con; ^*^P < 0.05, ***P < 0.001 vs. HN; ^△△△^*P* < 0.001 vs. Con+Mang.

Kidney fibrosis was assessed by Masson’s trichrome stain. Multiple blue-stained areas were observed in the tubular interstitium of kidneys from HN mice, indicating collagen accumulation. Mangiferin administration effectively reduced the renal interstitial fibrosis (**Figure 3A**). Similar result was observed in α-SMA staining (**Figure 3A**), as well as the change in α-SMA protein expression (**Figure 3B**). Furthermore, simultaneous treatment with mangiferin reversed the increased expression of fibronectin in kidneys of HN mice, further supporting the histological findings (**Figures 3B–D**). Normal control mice showed no difference in histological staining or fibrosis-related protein expression with or without mangiferin administration. Activation of the PKCβ pathway is thought to contribute to renal fibrosis in diabetic nephropathy (Toyoda et al., 2004). We also found increased expression of p-PKCβ in the HN model of our mice, which was reduced by mangiferin (**Figures 3B, E**), suggesting that therapeutic effect of mangiferin on fibrosis is at least in part mediated *via* modulation of PKCβ activation.

### Mangiferin Promoted Uric Acid Excretion Through Urine in HN Mice

**Figure 4:**
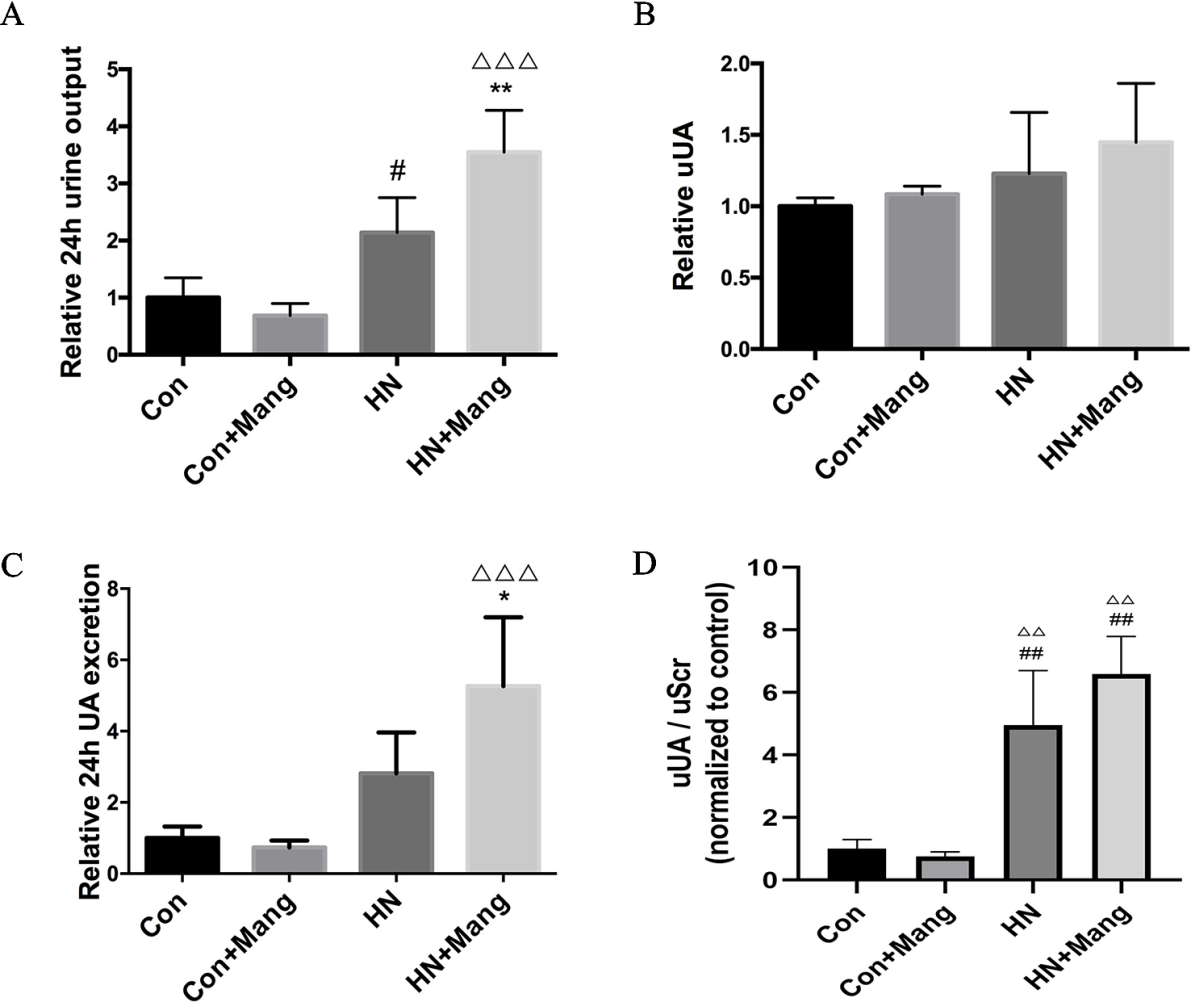
Effect of mangiferin on urinary uric acid excretion. The 24-hour urine output **(A)** and urine uric acid level was determined **(B)** from collected 24-hour urine. The 24-hour urine excretion was calculated **(C)**. Urine uric acid normalized by urine creatinine (uUA/uScr) was calculated **(D)**. Data was presented as normalized ratio comparing to control group. n = 3-6. ^#^*P* < 0.05 vs. Con; ^##^*P* < 0.01 vs. Con; ^*^P < 0.05, **P < 0.01 vs. HN; ^△△^*P* < 0.01 vs. Con+Mang; ^△△△^*P* < 0.001 vs. Con+Mang.

To further investigate the mechanisms contributing to renoprotection following mangiferin treatment in HN mice, we focused on uric acid generation and its renal excretion. Urine output was measured by collecting 24 h urine from metabolic cages. Increased urine output was found in the HN group, compared with control mice, but it was also found significantly higher in the HN group with simultaneous mangiferin treatment, compared with HN group (**Figure 4A**).

To figure out whether the increasing urine volume contributed to a rise in urinary uric acid excretion, uric acid levels were measured in the collected urine samples. There was no significant difference in urine uric acid levels among the four groups, albeit a slight rise was observed in the HN groups compared to control mice (**Figure 4B**). Calculated 24 h urine uric acid excretion was non-significantly higher in the HN group compared with control mice (p = 0.11), but markedly increased in HN mice treated with mangiferin compared to HN mice (**Figure 4C**). Normal mice with only mangiferin administration showed similar urinary uric acid excretion and urine output compared to normal control mice. Urine uric acid level normalized by urine creatinine (uUA/uScr) was calculated to evaluate uric acid excretion independent of urine volume. As shown in **Figure 4D**, there is significant increase of uUA/uScr level in HN and HN+Mang group compared to control group. However, no significant difference of uUA/uScr level was observed among HN mice with or without mangiferin treatment. Our result indicated that total urinary uric acid excretion was increased in HN mice, and further enhanced by mangiferin, which was mainly due to the increase of urine output.

### Mangiferin Elevating Uric Acid Excretion Was AQP2-Related While Urate Transporter-Independent

**Figure 5:**
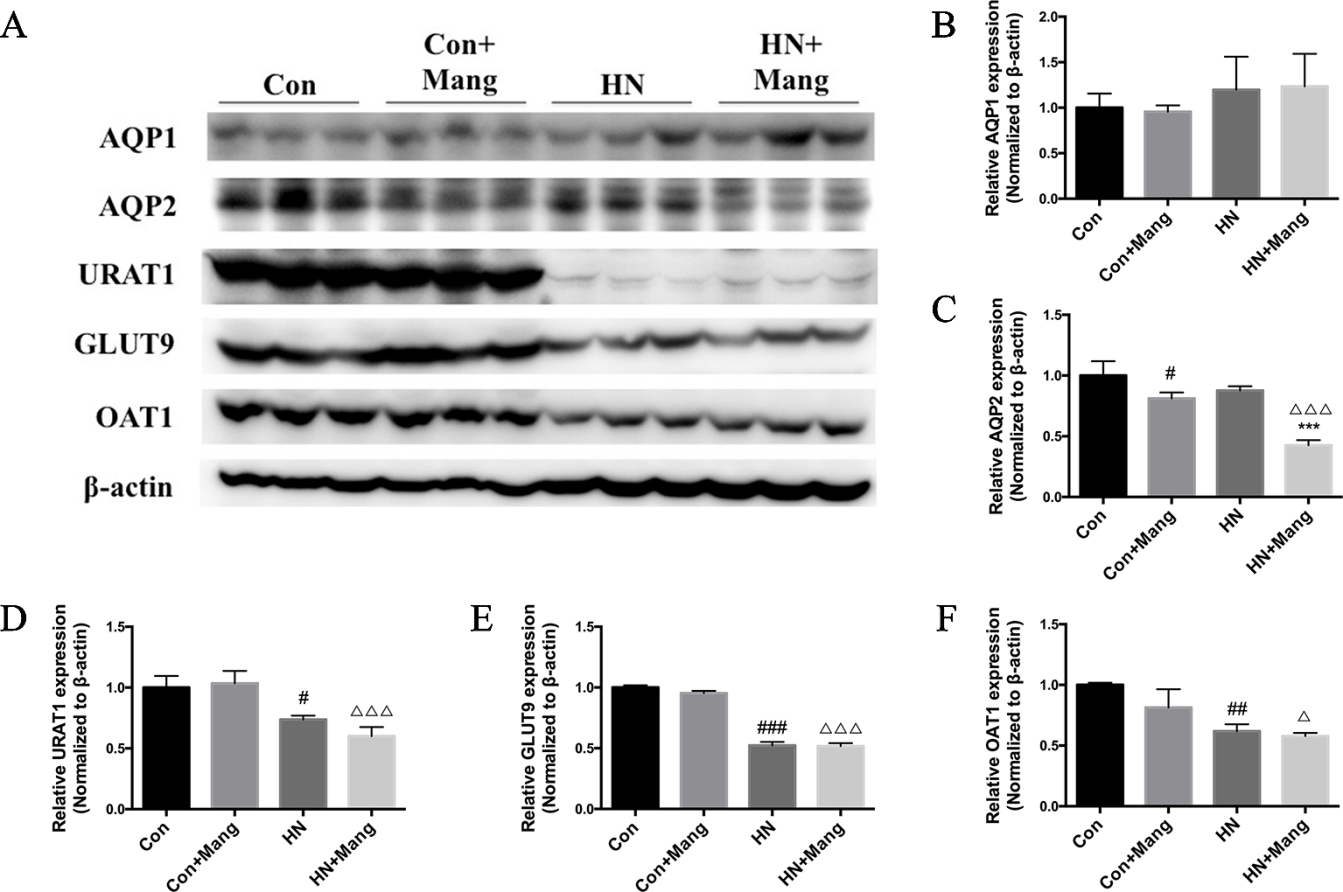
Effect of mangiferin on uric acid excretion-related transporters. Expression of AQP1, AQP2, URAT1, Glut9, and OAT1 was determined by western blot **(A)** and quantifications **(B–F)**. ^#^*P* < 0.05, ^##^*P* < 0.01, ^###^*P* < 0.001 vs. Con; ***P < 0.001 vs. HN; ^△^*P* < 0.05, ^△△△^*P* < 0.001 vs. Con+Mang.

Given the elevated 24 h urinary volume and 24 h uric acid excretion observed in mangiferin-treated HN mice, we next investigated possible underlying mechanism. Aquaporin 1 (AQP1) and aquaporin 2 (AQP2) are two major water channels located mainly on the apical membrane of proximal and collecting tubular cells, respectively. These channels mediate water reabsorption from the lumen to interstitium, thus modulating the urine output. We detected the expression level of AQP1 and AQP2 in kidneys (**Figure 5A**). AQP1 expression was not significantly different among the four groups (**Figure 5B**). However, AQP2 expression was significantly reduced in HN mice with mangiferin treatment, compared with HN mice (**Figure 5C**). Of note, mice treated with only mangiferin also showed significant decrease of AQP2 expression compared to normal control mice, indicating a direct influence of mangiferin on AQP2, independent of urate.

The expression of major urate transporters (i.e. URAT1, GLUT9, OAT1) in the kidney was also examined by the western blot (**Figure 5A**). These transporters were all down-regulated in HN mice, and was not affected by mangaferin treatment (**Figures 5D–F**). Therefore, decreased expression of urate transporters might contribute to the slight rise of uric acid excretion in HN mice, but was not influenced by mangiferin. It was rather the down-regulation of AQP2 that finally contributed to the increased uric acid excretion in mangiferin treated HN mice.

### Mangiferin Normalized XO Activity in HN Mice

**Figure 6:**
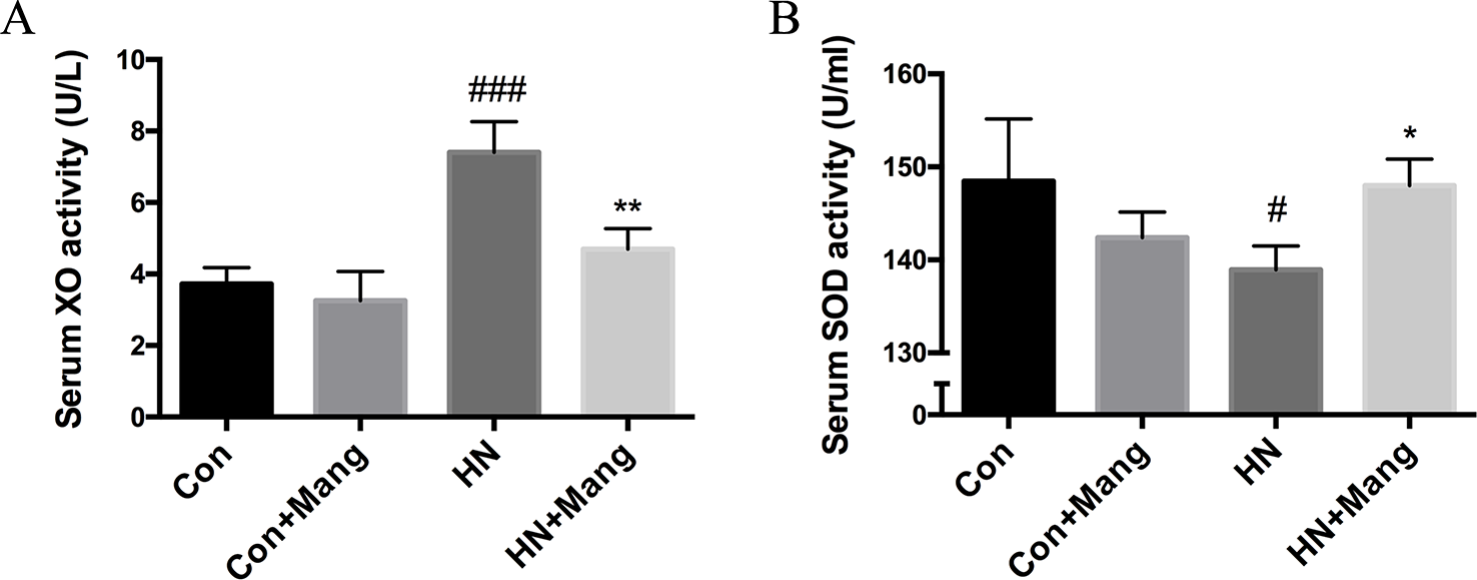
Effect of mangiferin on XOD activity and level of SOD. Serum XO **(A)** and SOD **(B)** activity was measured. n = 3-6. ^#^*P* < 0.05, ^###^*P* < 0.001 vs. Con; ^*^P < 0.05, **P < 0.01 vs. HN.

In addition to uric acid excretion, we also explored the effect of mangiferin on uric acid generation in this study. The activity of XO, a key enzyme catalyzing uric acid production, was assessed. Mangiferin attenuated the increased serum XO activity in HN mice (**Figure 6A**). Both normal and HN mice exhibited lower kidney XO activity with mangiferin administration (**Figure S1A**), which further confirmed the XO inhibitive effect of mangiferin specifically in the kidney. The inhibition rate of 100 μM mangiferin on XO activity *in vitro* was less than 40% (**Figure S2**), whereas 0.04 μM febuxostat, the clinical anti-hyperuricemic XO inhibitor, reached the inhibition rate of 95.4%.

Given that ROS (i.e. superoxide and hydrogen peroxide) are generated as by-products during XO-catalyzed reaction of hypoxanthine to xanthine and further to uric acid, it is predicted that mangiferin may exhibit some antioxidative properties by inhibiting XO activity. We also measured the activity of SOD in serum and kidney, one of the key antioxidant defense systems to eliminate ROS. Interestingly, SOD activity in serum was slightly but significantly decreased in HN mice, and this was reversed by mangiferin treatment (**Figure 6B**). Nevertheless, significantly higher kidney SOD activity was observed in HN mice without mangiferin treatment compared to normal mice (**Figure S1B**), which was the opposite of its change in the serum. There was no significant difference among the four groups of mice in kidney MDA activity (**Figure S1C**). HN mice did not exhibit higher MDA activity in kidney, which was supposed to be related to its high kidney SOD level.

Taken together, our results demonstrated an inhibitory effect of mangiferin on XO activity and modulation of serum SOD *in vivo*, which together contributed to less uric acid generation and potential reduction of oxidative stress.

## Discussion

In recent years, it has been found in basic research that hyperuricemia induced oxidative stress and compromised eNOS function, leading to renal endothelial dysfunction (Sanchez-Lozada et al., 2012). In addition, hyperuricemia-associated activation of the renin-angiotensin system promoted vascular intima proliferation, causing hypoperfusion of the renal tubules, and induced renal inflammation and fibrosis, contributing to HN (Kang et al., 2002; Jalal et al., 2011). In this study, we established a hyperuricemic nephropathy (HN) model in mice (**Figure 1**), which recapitulates many of the features characterizing this pathological condition (e.g. elevated serum uric acid level, impaired kidney function, renal injuries and fibrosis, as well as infiltration of inflammatory cells).

Mangiferin has been found protective in several diseases, including diabetic nephropathy (Li et al., 2010), cardiomyopathy (Hou et al., 2016), sepsis-induced acute kidney injury (He et al., 2014), and NAFLD (Wang et al., 2017). Among the underlying mechanisms, anti-inflammatory, anti-fibrotic, and antioxidative effects of mangiferin have been suggested to importantly contribute to its therapeutic effects. In this study, our results showed that mangiferin treatment effectively ameliorated both hyperuricemia and renal injury in the HN model, as evident from improved kidney index, normalized serum creatinine, and BUN levels. Moreover, the positive F4/80 and Masson’s trichrome staining of the kidney was significantly reduced by mangiferin, indicating also anti-inflammatory and anti-fibrotic effects of mangiferin in the HN model.

Hyperuricemia has been reported to induce renal inflammation through multiple mechanisms, including activation of JNK signaling pathway (Nomura et al., 2013) and NLRP3 inflammasome (Braga et al., 2017). JNK signaling pathway was closely associated with macrophage infiltration in distinct kidney injury models (de Borst et al., 2009; Tesch et al., 2016). NLRP3 inflammasome was activated in tubulointerstitial and glomerular diseases, promoting proinflammatory cytokines IL-1β and IL-18 production and excretion, causing renal inflammatory injuries (Chang et al., 2014). Our data showed substantial macrophage infiltration in glomeruli and tubulointerstitium of kidneys from HN mice, which was ameliorated by mangiferin. Furthermore, mangiferin prevented HN-induced activation of both JNK and NLRP3 in the kidney, which supports an anti-inflammatory effect of mangiferin also in this model. Nevertheless, due to the urate lowering and anti-inflammatory effects of mangiferin in other diseases (Niu et al., 2012; He et al., 2014; Wang et al., 2017), mangiferin was speculated to improve kidney inflammation by both decreasing uric acid level and direct inhibition of immune cell activation and infiltration.

In diabetes, hyperglycemia-associated activation of the PKCβ-MAPK pathway led to collagen accumulation and TGFβ-mediated extracellular matrix deposition, contributing to progressive renal fibrosis (Toyoda et al., 2004). Specific PKCβ inhibitors, such as ruboxistaurin, have been developed to prevent or treat diabetic nephropathy (Tuttle et al., 2005). Furthermore, the effects of PKCβ inhibition have also been investigated in models of non-diabetic progressive nephropathy. For example, in rats with subtotal nephrectomy, PKCβ inhibition with ruboxistaurin, significantly reduced TGFβ-mediated kidney fibrosis (Kelly et al., 2009). In our study, renal fibrosis in tubulointerstitial areas of HN kidney was associated with elevated fibronectin, α-SMA, and PKCβ expression, which were all prevented by mangiferin treatment. Taken together, it is proposed that mangiferin may alleviate kidney fibrosis *via* mechanisms that involve modulation of PKCβ activation.

It has been found in hyperuricemic rats that early urate-lowering therapy (ULT) using febuxostat and allopurinol both ameliorated kidney dysfunction (Sanchez-Lozada et al., 2005; Sanchez-Lozada et al., 2008a; Sanchez-Lozada et al., 2008b). A long-term quantitative study in gout patients also showed that decreased UA level by using febuxostat was correlated with eGFR improvement (Whelton et al., 2011). Therefore, lower uric acid level by mangiferin treatment was supposed to contribute to the kidney function improvement in HN mice. To further understand the mechanism(s) of mangiferin-mediated lowering of circulating uric acid, we focused on its role in modulating uric acid excretion and production.

Uricosuric drugs are usually known to promote uric acid excretion through urate transporters in hyperuricemia. The uricosurics benzbromarone and probenecid have been reported to inhibit GLUT9 and URAT1 (Hosoyamada et al., 2004; Anzai et al., 2008), and lesinurad inhibited URAT1 and OAT4 (Miner et al., 2016), leading to reduced urate reabsorption. However, reduction of urate reabsorption may cause substantial urate accumulation in the renal tubules before being excreted. This may increase the risk of uric acid crystallization and subsequent nephrotoxicity (Corrado et al., 2006). Unlike most uricosuric drugs, mangiferin promoted total urinary uric acid excretion mainly by elevating urine output, with no significant change in urine uric acid level. Increased uUA/uScr level in HN mice with or without mangiferin treatment indicated increased kidney uric acid excretion independent of urine volume (**Figure 4D**). This is in accordance with the decreased expression of urate transporters observed in these two groups (**Figure 5A**), which caused reduced reabsorption and elevated excretion of uric acid in kidney. uUA/uScr level among these two groups further confirmed that mangiferin did not affect urinary uric acid excretion when eliminating the factor of urine volume. The decreased urate transporters expression might be due to low levels of urate being filtered as a consequence of glomerular injuries (Nagura et al., 2016).

AQP2 channels, located primarily in the collecting duct, are usually activated by arginine vasopressin (AVP), leading to considerable water reabsorption (Christensen et al., 2000). In our study, we observed that mangiferin treatment reduced AQP2 expression in control kidneys and this effect was more pronounced in HN mice, and was accompanied with significantly higher urine output. As demonstrated in previous studies, variation of urine flow rate was positively related to the change of urate excretion (Diamond et al., 1972; Meisel and Diamond, 1976). However, the underlying mechanism(s) for this is not clear. Increased urate excretion by mangiferin treatment observed in HN mice in our study was also associated with increased urine flow rate, which was likely due to decreased AQP2 expression. Additionally, the significant decline of AQP2 expression in mangiferin-administrated control mice indicates a direct inhibitory effect of mangiferin on AQP2 regulation. Urine output in control mice with mangiferin treatment was not different from that in normal controls, which was probably adjusted through a compensatory mechanism. Finally, mangiferin did not affect AQP1 expression in any of the experimental groups.

As the key enzyme in catalyzing uric acid production, XO has been considered a classic drug target in anti-hyperuricemic treatment. Febuxostat and allopurinol are still first-line anti-hyperuricemic XO inhibitors. Previous report showed that norathyriol, a mangiferin metabolite *in vivo*, exerted XO inhibition and hypouricemic effects in mice (Niu et al., 2016). Consistently, we detected significantly decreased serum XO activity in HN mice treated with mangiferin. Apart from promoting uric acid excretion, the inhibitory effect on XO activity by mangiferin may also contribute to the lowering of serum uric acid in HN mice. Furthermore, as a by-product of the XO-catalyzed reaction, excessive ROS leads to oxidative stress with increased risk of several pathologies including kidney dysfunction and injuries (Daenen et al., 2019). Based on our result, HN mice displayed reduced serum SOD but increased kidney SOD. Opposite SOD levels were also reported in previous research. P.K. Gupta group used STZ to induce oxidative stress in rats. However, distinct SOD activities were found between heart and kidney (Muruganandan et al., 2002). Here, we speculated that a compensatory antioxidative mechanism occurred in the kidneys of HN mice, while the antioxidative defense was in general decreased in the systemic circulation and reversed by mangiferin.

Genetic variants of urate transporters such as ABCG2, URAT1, GLUT9 in human are a common cause of clinical hyperuricemia, which is harder to control and progress easily (Woodward et al., 2009; George and Keenan, 2013; Woodward, 2015). Given the multi-target therapeutic effects of mangiferin on HN independent of urate transporters, it is predicted that mangiferin might play a better role in the urate transporter dysfunction-related HN models. Loss of ABCG2 function caused urate overload in mice kidney (Matsuo et al., 2014). Future studies using ABCG2 Q140K mouse model will be of great interest and have clinical significance (Woodward and Hoque, 2017).

**Figure 7:**
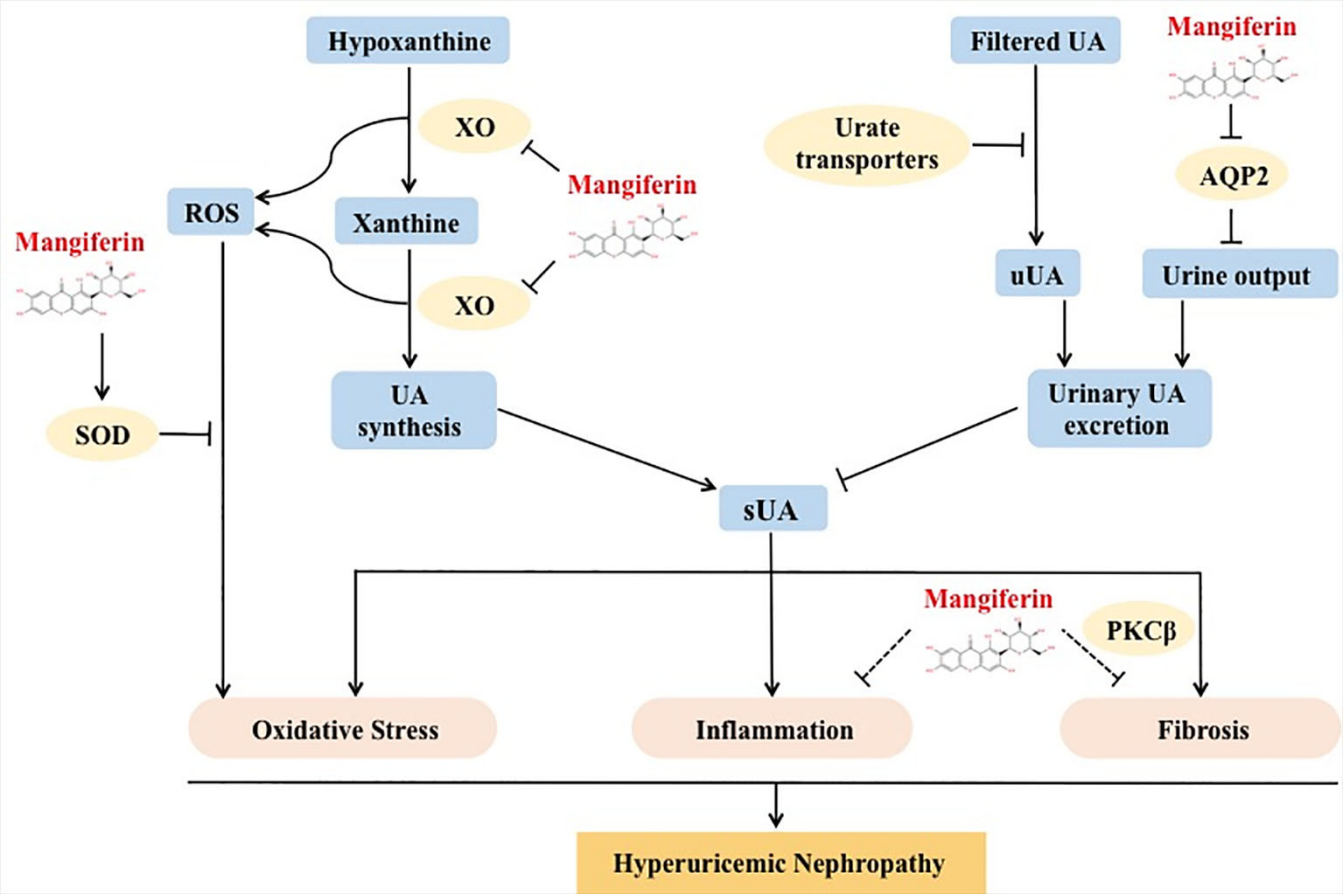
Multitarget role of mangiferin in protecting from HN. Mangiferin decreased uric acid level by inhibiting XO-mediated uric acid synthesis and promoting AQP2-related uric acid excretion. The decreasing level of uric acid or mangiferin or both contributed to anti-oxidative, anti-inflammatory and anti-fibrotic effect and prevented from HN.

To summarize, our study revealed a novel mechanism whereby mangiferin reduced serum urate levels by promoting urate renal excretion *via* inhibition of AQP2-dependent water reabsorption. Mangiferin suppressed renal injuries, inflammation, and fibrosis in a model of hyperuricemic nephropathy (HN), induced by oteracil potassium and hypoxanthine. The mechanism is proposed to be dual *via* a urate lowering effect and modulation of anti-inflammatory, anti-fibrotic, and antioxidative pathways (**Figure 7**). Our findings suggest that mangiferin might be a novel multi-target candidate to treat HN, with lower risk for kidney toxicity caused by highly concentrated uric acid in the renal tubules before excreted.

## Data Availability Statement

All datasets generated for this study are included in the article/**Supplementary Material**.

## Ethics Statement

This study was carried out in accordance with the guidelines established by the National Institutes of Health for the care and use of laboratory animals. The protocol was approved by the regional Animal Care Committee of CAMS & PUMC.

## Author Contributions

XL and FY conceived and designed research. XL, ZY, JT, XZ, and WZ performed the experiments. XL and FY analyzed the data. XL and FY interpreted the results of the experiments. XL prepared the figures. XL drafted the manuscript. MC, SW, and FY edited and revised the manuscript. FY approved the final version of the manuscript.

## Funding

This work was supported by the National Natural Science Foundation of China (No. 81600546) and CAMS Innovation Fund for Medical Sciences (CIFMS-2016-I2M-3-012).

## Conflict of Interest

The authors declare that the research was conducted in the absence of any commercial or financial relationships that could be construed as a potential conflict of interest.

## Supplementary Material

Figure S1 Effect of mangiferin on kidney XO, SOD and MDA activity. Kidney XO **(A)**, SOD **(B)**, and MDA **(C)** activity were measured. n = 8-10. ^###^*P* < 0.001 vs. Con.

Figure S2 Inhibitive effect of mangiferin on XO activity *in vitro*. Inhibition rates of XO activity by 100 μM mangiferin and 0.04 μM febuxostat were measured. n = 3-6.

## References

[B46] Anzai N. Ichida K. Jutabha P. Kimura T. Babu E. Jin C. J. (2008). Plasma urate level is directly regulated by a voltage-driven urate efflux transporter URATv1 (SLC2A9) in humans. J. Biol. Chem. 283, 26834–26838. 10.1074/jbc.C800156200 18701466

[B8] Boban M. Kocic G. Radenkovic S. Pavlovic R. Cvetkovic T. Deljanin-Ilic M. (2014). Circulating purine compounds, uric acid, and xanthine oxidase/dehydrogenase relationship in essential hypertension and end stage renal disease. Renal Fail. 36, 613–618. 10.3109/0886022X.2014.882240 24502620

[B36] Braga T. T. Forni M. F. Correa-Costa M. Ramos R. N. Barbuto J. A. Branco P. (2017). Soluble Uric Acid Activates the NLRP3 Inflammasome. Sci. Rep. 7, 39884. 10.1038/srep39884 28084303 PMC5233987

[B39] Chang A. Ko K. Clark M. R. (2014). The emerging role of the inflammasome in kidney diseases. Curr. Opin. Nephrol. Hypertens. 23, 204–210. 10.1097/01.mnh.0000444814.49755.90 24685591 PMC4189828

[B50] Christensen B. M. Zelenina M. Aperia A. Nielsen S. (2000). Localization and regulation of PKA-phosphorylated AQP2 in response to V(2)-receptor agonist/antagonist treatment. Am. J. Physiol. Renal Physiol. 278, F29–F42. 10.1152/ajprenal.2000.278.1.F29 10644653

[B3] Ciarla S. Struglia M. Giorgini P. Striuli R. Necozione S. Properzi G. (2014). Serum uric acid levels and metabolic syndrome. Arch. Physiol. Biochem. 120, 119–122. 10.3109/13813455.2014.924145 24914748

[B48] Corrado A. D'Onofrio F. Santoro N. Melillo N. Cantatore F. P. (2006). Pathogenesis, clinical findings and management of acute and chronic gout. Minerva Med. 97, 495–509. 17213786

[B53] Daenen K. Andries A. Mekahli D. Van Schepdael A. Jouret F. Bammens B. (2019). Oxidative stress in chronic kidney disease. Pediatr. Nephrol. 34, 975–991 10.1007/s00467-018-4005-4 30105414

[B37] de Borst M. H. Prakash J. Sandovici M. Klok P. A. Hamming I. Kok R. J. (2009). c-Jun NH2-terminal kinase is crucially involved in renal tubulo-interstitial inflammation. J. Pharmacol. Exp. Ther. 331, 896–905. 10.1124/jpet.109.154179 19717791

[B10] de Oliveira E. P. Burini R. C. (2012). High plasma uric acid concentration: causes and consequences. Diabetol. Metab. Syndr. 4, 12. 10.1186/1758-5996-4-12 22475652 PMC3359272

[B51] Diamond H. S. Lazarus R. Kaplan D. Halberstam D. (1972). Effect of urine flow rate on uric acid excretion in man. Arthritis Rheum. 15, 338–346. 10.1002/art.1780150403 5046465

[B2] Feig D. I. Kang D. H. Johnson R. J. (2008). Uric acid and cardiovascular risk. New Engl. J. Med. 359, 1811–1821. 10.1056/NEJMra0800885 18946066 PMC2684330

[B55] George R. L. Keenan R. T. (2013). Genetics of hyperuricemia and gout: implications for the present and future. Curr. Rheumatol. Rep. 15, 309. 10.1007/s11926-012-0309-8 23307580

[B7] Harrison R. (2004). Physiological roles of xanthine oxidoreductase. Drug Metab. Rev. 36, 363–375. 10.1081/DMR-120037569 15237859

[B25] He L. Peng X. Zhu J. Chen X. Liu H. Tang C. (2014). Mangiferin attenuate sepsis-induced acute kidney injury *via* antioxidant and anti-inflammatory effects. Am. J. Nephrol. 40, 441–450. 10.1159/000369220 25427663

[B11] Hosoyamada M. Ichida K. Enomoto A. Hosoya T. Endou H. (2004). Function and localization of urate transporter 1 in mouse kidney. J. Am. Soc. Nephrol.: JASN 15, 261–268. 10.1097/01.ASN.0000107560.80107.19 14747372

[B34] Hou J. Zheng D. Fung G. Deng H. Chen L. Liang J. (2016). Mangiferin suppressed advanced glycation end products (AGEs) through NF-kappaB deactivation and displayed anti-inflammatory effects in streptozotocin and high fat diet-diabetic cardiomyopathy rats. Can. J. Physiol. Pharmacol. 94, 332–340. 10.1139/cjpp-2015-0073 26751764

[B9] Ichida K. Matsuo H. Takada T. Nakayama A. Murakami K. Shimizu T. (2012). Decreased extra-renal urate excretion is a common cause of hyperuricemia. Nat. Commun. 3, 764. 10.1038/ncomms1756 22473008 PMC3337984

[B32] Jalal D. I. Maahs D. M. Hovind P. Nakagawa T. (2011). Uric acid as a mediator of diabetic nephropathy. Semin. Nephrol. 31, 459–465. 10.1016/j.semnephrol.2011.08.011 22000654 PMC3197214

[B4] Jalal D. I. Chonchol M. Chen W. Targher G. (2013). Uric acid as a target of therapy in CKD. Am. J. Kidney Dis. 61, 134–146. 10.1053/j.ajkd.2012.07.021 23058478 PMC3525781

[B5] Johnson R. J. Nakagawa T. Jalal D. Sanchez-Lozada L. G. Kang D. H. Ritz E. (2013). Uric acid and chronic kidney disease: which is chasing which? Nephrol. Dial. Transplant. 28, 2221–2228. 10.1093/ndt/gft029 23543594 PMC4318947

[B6] Jordan D. M. Choi H. K. Verbanck M. Topless R. Won H. H. Nadkarni G. (2019). No causal effects of serum urate levels on the risk of chronic kidney disease: a Mendelian randomization study. PloS Med. 16, e1002725. 10.1371/journal.pmed.1002725 30645594 PMC6333326

[B31] Kang D. H. Nakagawa T. Feng L. Watanabe S. Han L. Mazzali M. (2002). A role for uric acid in the progression of renal disease. J. Am. Soc. Nephrol. 13, 2888–2897. 10.1097/01.ASN.0000034910.58454.FD 12444207

[B16] Kaufmann P. Torok M. Hanni A. Roberts P. Gasser R. Krahenbuhl S. (2005). Mechanisms of benzarone and benzbromarone-induced hepatic toxicity. Hepatology 41, 925–935. 10.1002/hep.20634 15799034

[B41] Kelly D. J. Edgley A. J. Zhang Y. Thai K. Tan S. M. Cox A. J. (2009). Protein kinase C-beta inhibition attenuates the progression of nephropathy in non-diabetic kidney disease. Nephrol. Dial. Transplant. 24, 1782–1790. 10.1093/ndt/gfn729 19155535

[B17] Lee M. H. Graham G. G. Williams K. M. Day R. O. (2008). A benefit-risk assessment of benzbromarone in the treatment of gout Was its withdrawal market In best interest patients? Drug Saf. 31, 643–665. 10.2165/00002018-200831080-00002 18636784

[B33] Li X. Cui X. Sun X. Li X. Zhu Q. Li W. (2010). Mangiferin prevents diabetic nephropathy progression in streptozotocin-induced diabetic rats. Phytother. Res. 24, 893–899. 10.1002/ptr.3045 19960420

[B58] Matsuo H. Nakayama A. Sakiyama M. Chiba T. Shimizu S. Kawamura Y. (2014). ABCG2 dysfunction causes hyperuricemia due to both renal urate underexcretion and renal urate overload. Sci. Rep. 4, 3755. 10.1038/srep03755 24441388 PMC3895923

[B52] Meisel A. Diamond H. (1976). Effect of vasopressin on uric acid excretion: evidence for distal nephron reabsorption of urate in man. Clin. Sci. Mol. Med. 51, 33–40. 10.1042/cs0510033 939064

[B18] Melethil S. Conway W. D. (1976). Urinary excretion of probenecid and its metabolites in humans as a function of dose. J. Pharm. Sci. 65, 861–865. 10.1002/jps.2600650615 6781

[B28] Meng X. Mao Z. Li X. Zhong D. Li M. Jia Y. (2017). Baicalein decreases uric acid and prevents hyperuricemic nephropathy in mice. Oncotarget 8, 40305–40317. 10.18632/oncotarget.16928 28445133 PMC5522264

[B47] Miner J. N. Tan P. K. Hyndman D. Liu S. Iverson C. Nanavati P. (2016). Lesinurad, a novel, oral compound for gout, acts to decrease serum uric acid through inhibition of urate transporters in the kidney. Arthritis Res. Ther. 18, 214. 10.1186/s13075-016-1107-x 27716403 PMC5048659

[B54] Muruganandan S. Gupta S. Kataria M. Lal J. Gupta P. K. (2002). Mangiferin protects the streptozotocin-induced oxidative damage to cardiac and renal tissues in rats. Toxicology 176, 165–173. 10.1016/S0300-483X(02)00069-0 12093613

[B49] Nagura M. Tamura Y. Kumagai T. Hosoyamada M. Uchida S. (2016). Uric acid metabolism of kidney and intestine in a rat model of chronic kidney disease. Nucleosides Nucleotides Nucleic Acids 35, 550–558. 10.1080/15257770.2016.1163379 27906625

[B13] Nigam S. K. Bush K. T. Martovetsky G. Ahn S. Y. Liu H. C. Richard E. (2015). The organic anion transporter (OAT) family: a systems biology perspective. Physiol. Rev. 95, 83–123. 10.1152/physrev.00025.2013 25540139 PMC4281586

[B23] Niu Y. Lu W. Gao L. Lin H. Liu X. Li L. (2012). Reducing effect of mangiferin on serum uric acid levels in mice. Pharm. Biol. 50, 1177–1182. 10.3109/13880209.2012.663763 22881143

[B27] Niu Y. Liu J. Liu H. Y. Gao L. H. Feng G. H. Liu X. (2016). Hypouricaemic action of mangiferin results from metabolite norathyriol *via* inhibiting xanthine oxidase activity. Pharm. Biol. 54, 1680–1686. 10.3109/13880209.2015.1120322 26916555

[B35] Nomura J. Busso N. Ives A. Tsujimoto S. Tamura M. So A. (2013). Febuxostat, an inhibitor of xanthine oxidase, suppresses lipopolysaccharide-induced MCP-1 production *via* MAPK phosphatase-1-mediated inactivation of JNK. PloS One 8, e75527. 10.1371/journal.pone.0075527 24086554 PMC3783396

[B26] Pal P. B. Sinha K. Sil P. C. (2014). Mangiferin attenuates diabetic nephropathy by inhibiting oxidative stress mediated signaling cascade, TNFalpha related and mitochondrial dependent apoptotic pathways in streptozotocin-induced diabetic rats. PloS One 9, e107220. 10.1371/journal.pone.0107220 25233093 PMC4169432

[B19] Pan Y. Kong L. D. (2016). Urate transporter URAT1 inhibitors: a patent review (2012–2015). Expert Opin. Ther. Pat. 26, 1–10. 10.1080/13543776.2016.1213243 27414413

[B1] Richette P. Bardin T. (2010). Gout. Lancet 375, 318–328. 10.1016/S0140-6736(09)60883-7 19692116

[B42] Sanchez-Lozada L. G. Tapia E. Santamaria J. Avila-Casado C. Soto V. Nepomuceno T. (2005). Mild hyperuricemia induces vasoconstriction and maintains glomerular hypertension in normal and remnant kidney rats. Kidney Int. 67, 237–247. 10.1111/j.1523-1755.2005.00074.x 15610247

[B43] Sanchez-Lozada L. G. Tapia E. Soto V. Avila-Casado C. Franco M. Wessale J. L. (2008a). Effect of febuxostat on the progression of renal disease in 5/6 nephrectomy rats with and without hyperuricemia. Nephron Physiol. 108, 69–78. 10.1159/000127837 18434753

[B44] Sanchez-Lozada L. G. Tapia E. Soto V. Avila-Casado C. Franco M. Zhao L. (2008b). Treatment with the xanthine oxidase inhibitor febuxostat lowers uric acid and alleviates systemic and glomerular hypertension in experimental hyperuricaemia. Nephrol. Dial. Transplant. 23, 1179–1185. 10.1093/ndt/gfm783 18048425

[B30] Sanchez-Lozada L. G. Lanaspa M. A. Cristobal-Garcia M. Garcia-Arroyo F. Soto V. Cruz-Robles D. (2012). Uric acid-induced endothelial dysfunction is associated with mitochondrial alterations and decreased intracellular ATP concentrations. Nephron. Exp. Nephrol. 121, e71–e78. 10.1159/000345509 23235493 PMC3656428

[B20] Stamp L. K. Merriman T. R. Singh J. A. (2018). Expert opinion on emerging urate-lowering therapies. Expert Opin. Emerg Drugs 23, 201–209. 10.1080/14728214.2018.1527899 30244605

[B15] Tan P. K. Miner J. N. (2017). Uric acid transporter inhibitors for gout. ADMET DMPK 5, 16. 10.5599/admet.5.2.387

[B14] Tan P. K. Ostertag T. M. Miner J. N. (2016). Mechanism of high affinity inhibition of the human urate transporter URAT1. Sci. Rep. 6, 34995. 10.1038/srep34995 27713539 PMC5054527

[B21] Telang M. Dhulap S. Mandhare A. Hirwani R. (2013). Therapeutic and cosmetic applications of mangiferin: a patent review. Expert Opin. Ther. Pat. 23, 1561–1580. 10.1517/13543776.2013.836182 24066838

[B38] Tesch G. H. Ma F. Y. Nikolic-Paterson D. J. (2016). ASK1: a new therapeutic target for kidney disease. Am. J. Physiol. Renal Physiol. 311, F373–F381. 10.1152/ajprenal.00208.2016 27226108

[B29] Toyoda M. Suzuki D. Honma M. Uehara G. Sakai T. Umezono T. (2004). High expression of PKC-MAPK pathway mRNAs correlates with glomerular lesions in human diabetic nephropathy. Kidney Int. 66, 1107–1114. 10.1111/j.1523-1755.2004.00798.x 15327405

[B40] Tuttle K. R. Bakris G. L. Toto R. D. McGill J. B. Hu K. Anderson P. W. (2005). The effect of ruboxistaurin on nephropathy in type 2 diabetes. Diabetes Care 28, 2686–2690. 10.2337/diacare.28.11.2686 16249540

[B12] Vitart V. Rudan I. Hayward C. Gray N. K. Floyd J. Palmer C. N. (2008). SLC2A9 is a newly identified urate transporter influencing serum urate concentration, urate excretion and gout. Nat. Genet. 40, 437–442. 10.1038/ng.106 18327257

[B24] Wang H. Zhu Y. Y. Wang L. Teng T. Zhou M. Wang S. G. (2017). Mangiferin ameliorates fatty liver *via* modulation of autophagy and inflammation in high-fat-diet induced mice. Biomed. Pharmacother. 96, 328–335. 10.1016/j.biopha.2017.10.022 29024899

[B45] Whelton A. MacDonald P. A. Zhao L. Hunt B. Gunawardhana L. (2011). Renal function in gout long-term treatment effects of febuxostat. J. Clin. Rheumatol. 17, 7–13. 10.1097/RHU.0b013e318204aab4 21169856

[B59] Woodward O. Hoque K. M. (2017). New mouse model of gout risk variant, ABCG2 Q141K, reveals unexpectedly severe molecular and functional defect in ABCG2 mediated intestinal uric acid secretion [abstract]. Arthritis Rheumatol. 69 (suppl 10). https://acrabstracts.org/abstract/new-mouse-model-of-gout-risk-variant-abcg2-q141k-reveals-unexpectedly-severe-molecular-and-functional-defect-in-abcg2-mediated-intestinal-uric-acid-secretion/

[B57] Woodward O. M. Kottgen A. Coresh J. Boerwinkle E. Guggino W. B. Kottgen M. (2009). Identification of a urate transporter, ABCG2, with a common functional polymorphism causing gout. Proc. Natl. Acad. Sci. U. S. A. 106, 10338–10342. 10.1073/pnas.0901249106 19506252 PMC2700910

[B56] Woodward O. M. (2015). ABCG2: the molecular mechanisms of urate secretion and gout. Am. J. Physiol. Renal Physiol. 309, F485–F488. 10.1152/ajprenal.00242.2015 26136557 PMC4572392

[B22] Yang H. Gao L. Niu Y. Zhou Y. Lin H. Jiang J. (2015). Mangiferin Inhibits Renal Urate Reabsorption by Modulating Urate Transporters in Experimental Hyperuricemia. Biol. Pharm. Bull. 38, 1591–1598. 10.1248/bpb.b15-00402 26228630

