# Supplementary figures and images for: Mangiferin Ameliorates Hyperuricemic Nephropathy Which Is Associated With Downregulation of AQP2 and Increased Urinary Uric Acid Excretion
Source: Front Pharmacol. 2020 Feb 7;11:49. doi: 10.3389/fphar.2020.00049 (PMC7020245; doi:10.3389/fphar.2020.00049)

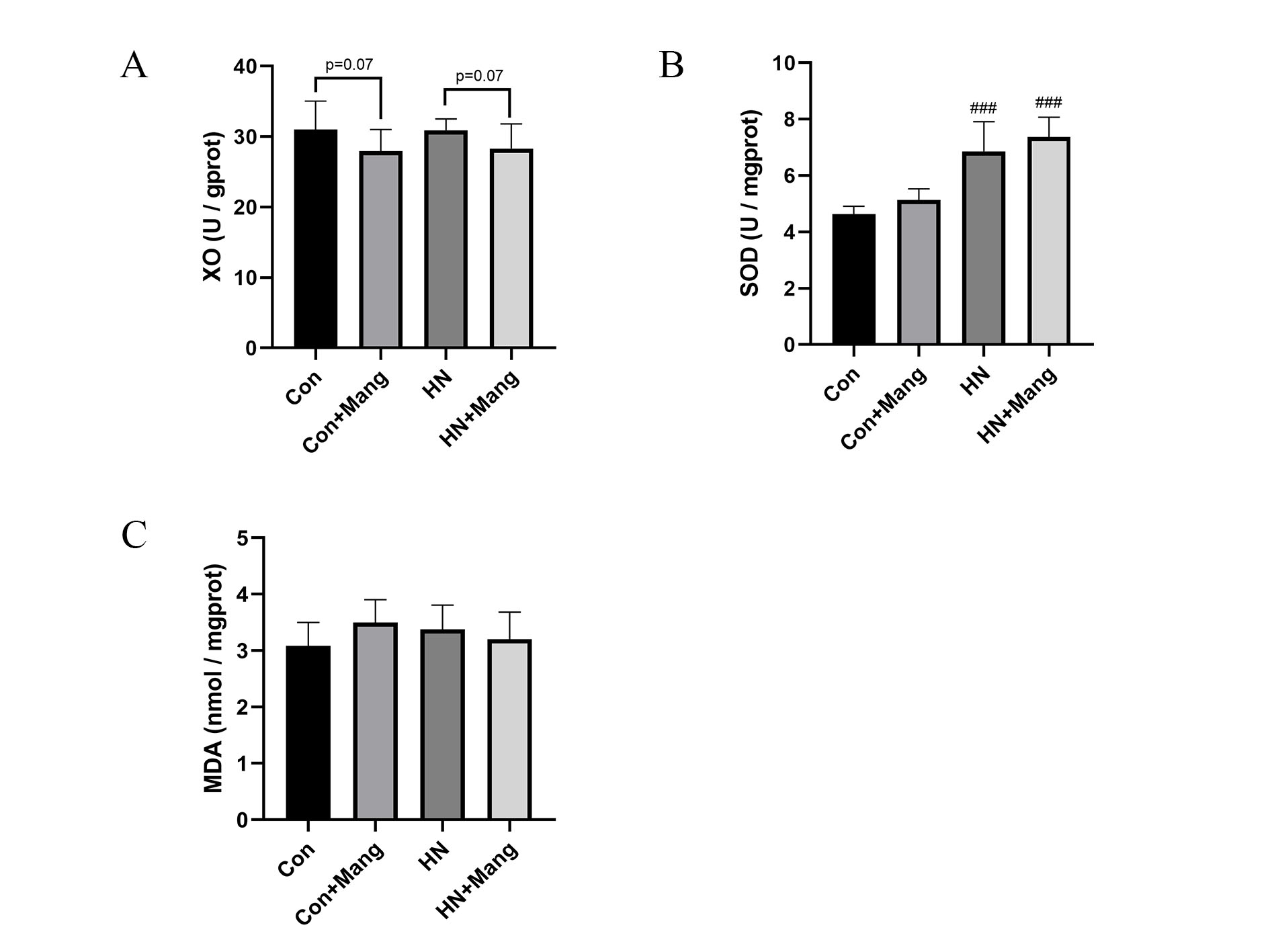

Supplement: Figure S1 — Effect of mangiferin on kidney XO, SOD and MDA activity. Kidney XO (A), SOD (B), and MDA (C) activity were measured. n = 8-10. ###P < 0.001 vs. Con. [file Image_1.jpeg]

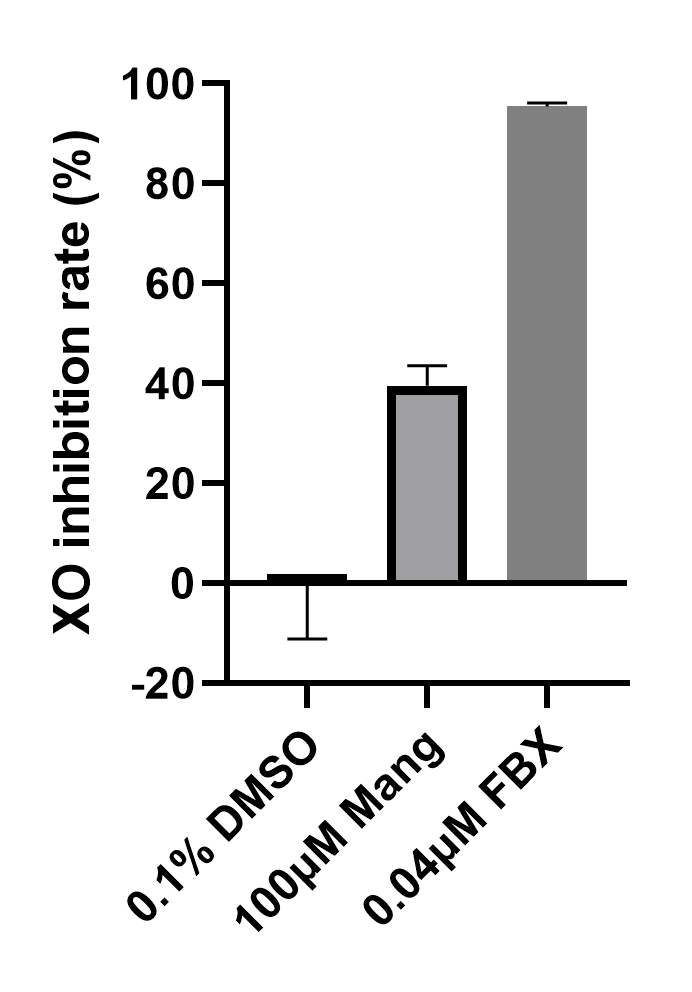

Supplement: Figure S2 — Inhibitive effect of mangiferin on XO activity in vitro. Inhibition rates of XO activity by 100 μM mangiferin and 0.04 μM febuxostat were measured. n = 3-6. [file Image_2.tif]
